# Biofilm Formation by Shiga Toxin-Producing *Escherichia coli* on Stainless Steel Coupons as Affected by Temperature and Incubation Time

**DOI:** 10.3390/microorganisms7040095

**Published:** 2019-03-31

**Authors:** Zhi Ma, Emmanuel W. Bumunang, Kim Stanford, Xiaomei Bie, Yan D. Niu, Tim A. McAllister

**Affiliations:** 1 College of Food Science and Technology, Nanjing Agriculture University, Nanjing 210095, China; mazhi19900504@sina.com; 2 Agriculture and Agri-Food Canada, Lethbridge, AB T1J 4B1, Canada; emmanuel.bumunang@canada.ca; 3 Alberta Agriculture and Forestry, Lethbridge, AB T1J 4V6, Canada; kim.stanford@gov.ab.ca; 4 Faculty of Veterinary Medicine, University of Calgary, Calgary, AB T2N 4Z6, Canada; dongyan.niu@ucalgary.ca

**Keywords:** Shiga toxin-producing *Escherichia coli* (STEC), biofilm formation, temperature, stainless steel

## Abstract

Forming biofilm is a strategy utilized by Shiga toxin-producing *Escherichia coli* (STEC) to survive and persist in food processing environments. We investigated the biofilm-forming potential of STEC strains from 10 clinically important serogroups on stainless steel at 22 °C or 13 °C after 24, 48, and 72 h of incubation. Results from crystal violet staining, plate counts, and scanning electron microscopy (SEM) identified a single isolate from each of the O113, O145, O91, O157, and O121 serogroups that was capable of forming strong or moderate biofilms on stainless steel at 22 °C. However, the biofilm-forming strength of these five strains was reduced when incubation time progressed. Moreover, we found that these strains formed a dense pellicle at the air-liquid interface on stainless steel, which suggests that oxygen was conducive to biofilm formation. At 13 °C, biofilm formation by these strains decreased (*P* < 0.05), but gradually increased over time. Overall, STEC biofilm formation was most prominent at 22 °C up to 24 h. The findings in this study identify the environmental conditions that may promote STEC biofilm formation in food processing facilities and suggest that the ability of specific strains to form biofilms contributes to their persistence within these environments.

## 1. Introduction

Currently, biofilm formation has gained considerable attention in food processing environments. The attachment of microorganisms and subsequent development of biofilms in these environments may be a leading cause of the adulteration of food, which results from the biofouling of pipelines and processing equipment [1]. In addition, biofilms of spoilage and pathogenic microflora that form on contact surfaces are often responsible for the contamination of food during post-processing production [2,3]. It has been shown that, even with diligent cleaning and sanitation, microorganisms within biofilms can remain viable on equipment surfaces [4].

Bacteria can readily bind to stainless steel and polymer surfaces in food production systems and form biofilms where cells are embedded within a matrix made up of proteins, carbohydrates, and extracellular DNA [5,6]. Portions of mature biofilm often detach and are able to colonize downstream environments [7]. Biofilm formation in food processing environments increases the resistance of cells to a number of stressors including starvation, heat, cold, and sanitizers [8,9].

Shiga toxin–producing *Escherichia coli* (STEC) are foodborne pathogens responsible for human enteric infections [10]. They are associated with important public health concerns worldwide. Symptoms associated with STEC infections range from abdominal cramps and bloody diarrhea to post-infection complications arising from hemolytic-uremic syndrome [11]. According to the Public Health Agency of Canada [12], more than 652 cases of STEC infections occur in Canada each year. The rate of STEC O157:H7 has remained relatively constant at 1.2 cases per 100,000 people per year since 2010. For STEC non-O157, the incidence rate increased slightly between 2010 and 2016 from 0.25 to 0.6 cases per 100,000 people per year. STEC O157:H7 is the most predominant serotype causing outbreaks, but other STEC serogroups, such as O26, O45, O91, O103, O111, O113, O121, O128, and O145, have also been linked to severe illness [13,14]. Although the first reported infections by STEC were associated with contaminated meat, foods such as cheese, vegetables, and drinking water have also been implicated in STEC outbreaks [15,16,17]. STEC isolates of different origins (i.e., animal, food, and human) can form strong biofilms on various food-contact surfaces [17]. The extracellular matrix of STEC biofilms mainly consists of proteins, poly-*N*-acetylglucosamine, cellulose, and colanic acid [5]. Although biofilm formation by STEC isolates is influenced by temperature and time [9], researchers have found that the number of STEC O157:H7 reached up to 5 log CFU/mL in beef juice at 4 °C over 72 h [6]. Thus, STEC biofilms are a potential threat to food hygiene and may become a source of infectious disease in both farm and food-processing environments.

Numerous studies have evaluated the impact of STEC O157 biofilms on food safety, as well as understanding the mechanisms and genetic basis for biofilm formation by this pathogen [18,19]. In contrast, there are relatively few reports on the ability of non-O157 STEC to form biofilms on food-contact surfaces. Adator et al. demonstrated that 12 non-O157 strains remained viable within dry-surface biofilms on stainless steel for at least 30 days and were able to contaminate fresh lettuce within 2 min of exposure [17]. Furthermore, Rong et al. showed that O26:H11 and O111:H8 exhibited a superior ability to form biofilms at the air-liquid interface on glass surfaces and be insensitive to sanitizers [20]. These studies demonstrated that non-O157 can adhere to food contact surfaces and subsequently result in contamination of vegetables and meat.

Thirty-six non-O157 STEC strains from nine serogroups (O113, O145, O91, O26, O121, O128, O103, O45, and O111) were investigated for biofilm formation on polystyrene in our previous study [21]. Of these strains, EC20020170 O113:H21, EC19990166 O145:H25, EC20010076 O91:H21, EC19970119 O26:H11, EC19990161 O121:H19, EC19960949 O128:NM, EC19970327 O103:H2, EC19940040 O45:H2, and EC20030053 O111:NM from each serogroup formed strong biofilms on polystyrene at 22 °C and 37 °C [21]. This finding coupled with the previous studies motivated us to further explore their biofilm-forming abilities on stainless steel, since it is the most common contact surface used in food-processing plants. In addition, we included a representative O157 strain of phage type 14a, which is the predominant phage type isolated from humans in Canada. Therefore, the objective of this study was to investigate the biofilm forming potential of STEC over time on stainless steel surfaces at different temperatures.

## 2. Materials and Methods

### 2.1. Bacterial Strains and Cultivation

EC20020170 O113:H21, EC19990166 O145:H25, EC20010076 O91:H21, EC19970119 O26:H11, EC19990161 O121:H19, EC19960949 O128:NM, EC19970327 O103:H2, EC19940040 O45:H2, EC20030053 O111:NM, and EC2011007 O157:H7 were kindly provided by Dr. Roger Johnson of the Public Health Agency of Canada (Guelph, ON, USA). All strains were streaked onto Lysogeny broth (LB) agar (Sigma-Aldrich, Oakville, ON, USA) and incubated at 37 °C for 18 h. An isolated colony was then inoculated into 10 mL of Minimal Salt (M9) broth (Sigma-Aldrich) supplemented with 0.4% glucose, 0.02% MgSO_4_, and 0.001% CaCl_2_ (*w*/*v*) and grown at 37 °C, on a rocker platform at 180 rpm for 18 h.

### 2.2. Biofilm Formation

Type-304 stainless steel coupons (No. 2b finish, 2.54 cm × 7.62 cm × 0.081 cm, Biosurface, Bozeman, MT, USA) were used to assess biofilm formation. Prior to use, coupons were soaked in 10% bleach (0.5% hypochlorite) for 24 h. This was followed by rinsing three times with sterile distilled water to remove residual hypochlorite and then dried in the biosafety cabinet. Coupons were then treated with 70% ethanol and air-dried for 5 min at room temperature. Lastly, the coupons were autoclaved at 121 °C for 15 min.

To assess biofilm formation, a conventional static assay was used with minor modifications [22]. Briefly, overnight STEC cultures were diluted in M9 medium to achieve a final concentration of 7 log CFU/mL. Subsequently, 20 mL of the diluted cultures were introduced into 50 mL falcon tubes containing a sterile stainless steel coupon. The tubes were loosely capped and incubated at 13 °C or 22 °C for 24, 48, or 72 h at which point biofilm formation was assessed. Two replicate coupons for each strain were evaluated, and coupons in un-inoculated medium were used as negative controls. Data are presented as the average of three independent trials.

### 2.3. Crystal Violet Staining

Following incubation, the coupons were carefully removed from the growth medium using sterile forceps, gently tapped against the side of the falcon tube to remove excess liquid, and rinsed three times with 25 mL of sterile filtered water to remove loosely-adherent bacteria. Subsequently, the coupons with attached bacterial cells were fixed with 25-mL absolute methanol (analytical grade, >99%, Sigma-Aldrich) for 15 min. Coupons were air-dried for 2 min, and stained with 0.5 % (*w*/*v*) crystal violet solution (Sigma-Aldrich) for 15 min, which was followed by three washes with distilled water and air-drying. The dye bound to the biofilm was then dissolved with 25 mL of 33% glacial acetic acid (Sigma-Aldrich) and measured at a wavelength of 590 nm using a spectrophotometer as described previously [21].

### 2.4. Enumeration of the Planktonic and Attached Cells

To enumerate the planktonic cells after each period, 1 mL of the cell suspension was pipetted from the falcon tubes, serially diluted with 10 mM phosphate buffered saline (PBS, pH 7.4), plated on LB agar, and incubated at 37 °C for 18 h. To recover the attached bacterial cells, the coupons were rinsed three times with sterile water, immersed into 25 mL of sterile PBS, and sonicated at 20 kHz for 10 min. After sonication, the tubes containing coupons were vigorously vortexed for 1 min, and 1 mL of the bacterial suspension was serially diluted, plated on LB agar, and incubated at 37 °C for 18 h. Bacteria were enumerated and the results were expressed as the average of the data from three independent assays.

### 2.5. SEM Analysis

Based on the above assays, three strong biofilm formers (strains O113, O145 and O91) were further observed by scanning electron microscopy (SEM), as described previously [23]. Coupons with biofilms were rinsed three times as described above, air-dried, and then fixed in 2.5 % glutaraldehyde (*v*/*v*) for 24 h. Subsequently, the samples were dehydrated in a series of ethanol dilutions (*v*/*v*) (i.e., 10%, 30%, 50%, 70%, 90%, and 100%) and isobutyl alcohol dilutions (*v*/*v*) (i.e., 10%, 30%, 50%, 70%, 90%, and 100%). The samples were then treated with 100% (*v*/*v*) hexamethyldisilazane for 10 min and coated with gold using a sputter coater and visualized using a SEM (HITACHI S-4800, Japan).

### 2.6. Statistical Analysis

Results from biofilm formation were compiled from the three independent experiments. Data were reported as the averages of replicates ± the standard deviation. The student’s *t*-test and one-way ANOVA with the SPSS software (19.0, IBM, Armonk, NY, USA) were used to calculate *P* values among treatment groups. Significant differences were presented at a 95% confidence level (*P* ≤ 0.05).

## 3. Results

### 3.1. Growth of the Planktonic Cells in M9 Medium

As shown in Figure S1, all strains grew well and exhibited similar growth patterns at 22 °C, which reached the stationary phase within 24 h at about 9 log CFU/mL, but, at 13 °C, it required 72 h for cultures to reach an average of 8.5 log CFU/mL.

### 3.2. Biofilm Formation by STEC on a Stainless Steel Surface

**Figure 1:**
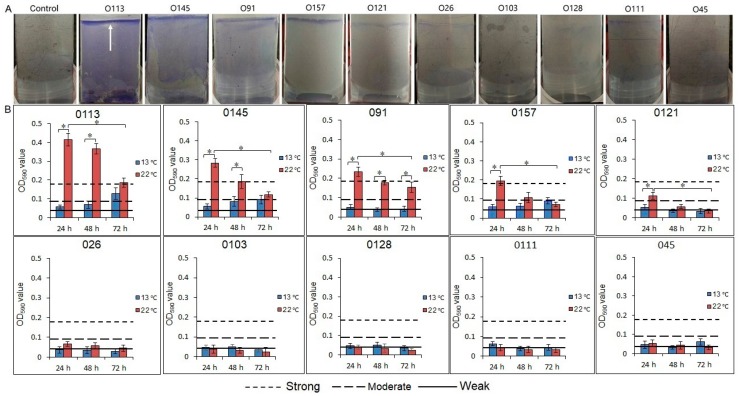
Biofilm formation of 10 STEC strains on a stainless steel surface. (**A**) Biofilms of 10 STEC strains were stained by crystal violet after incubation at 22 °C for 24 h. The arrow shows that some strains formed a dense pellicle at the air-liquid interface. (**B**) Biofilm formation of 10 STEC strains in M9 medium at 22 °C or 13 °C after 24, 48, and 72 h. The vertical axis represents the average of OD values, determined at 590 nm. Horizontal lines represent the cutoff values between weak, moderate, and strong biofilm producers. The cutoff optical density value (ODc) of 0.043 is defined as three standard deviations above the mean OD of the negative controls. Strains were classified as OD ≤ ODc (0.043), no biofilm producer, ODc < OD ≤ 2 × ODc, weak biofilm producers, 2 × ODc < OD ≤ 4 × ODc, moderate biofilm producers, and 4 × ODc < OD, strong biofilm producers. OD, optical density, STEC, Shiga toxin-producing *E. coli*. Asterisk denotes significant difference (*P* < 0.05).

The STEC isolates differed in their ability to form biofilms on stainless steel (Figure 1A). Based on the OD_590nm_ produced by biofilms, strains were classified as no biofilm, weak, moderate, or strong biofilm producers, as previously described [24]. The cutoff optical density value (ODc) of 0.043 was three standard deviations above the mean OD of negative controls. Isolates were classified as no (A_590_ < 0.043), weak (0.086 > A_590_ > 0. 043), moderate (0.172 > A_590_ > 0.086), or strong biofilm formers (A_590_ > 0.172; Figure 1B). At 22 °C, O113 exhibited the highest biofilm-forming capacity (*P* < 0.05), followed by O145, O91, O157, and O121, respectively. The isolates from serogroups O26, O103, O128, O111, and O45 formed only weak biofilms. We also found that the attached biomass of strains O113, O145, O91, O157, and O121 decreased (*P* < 0.05) with incubation time at 22 °C (Figure 1B). Compared to 22 °C, the biofilm formation of isolates from serogroups O113, O145, O91, O157, and O121 decreased (*P* < 0.05) at 13 °C. Only the isolate from serogroup O113 formed a moderate biofilm at 13 °C after 72 h of incubation.

### 3.3. Enumeration of the Biofilm Cells

**Figure 2:**
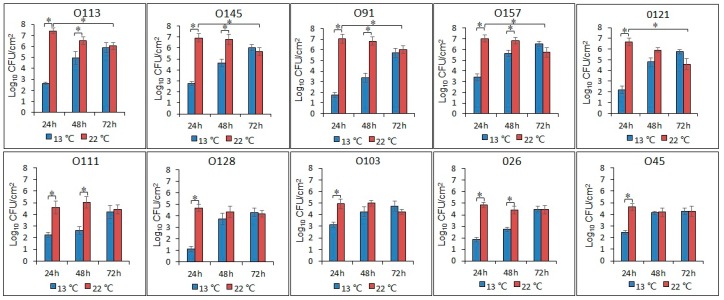
Number of biofilm cells of the 10 STEC isolates after incubation at 22 °C and 13 °C for 24, 48, and 72 h. Asterisk denotes a significant difference (*P* < 0.05).

Populations of biofilm cells of strains O113, O145, O91, O157, and O121 all reached approximately 7.0 log CFU/cm^2^ at 22 °C after 24 h, but consistently decreased (*P* < 0.05) to less than 5.9 log CFU/cm^2^ by 72 h (Figure 2). The number of biofilm cells for these five isolates ranged from 2.0 to 3.3 log CFU/cm^2^ at 13 °C at 24 h, achieving 5.5 to 6.4 log CFU/cm^2^ over 72 h. In contrast, there was no difference in the number of biofilm cells of O111, O128, O103, O26, and O45 at 22 °C, with the concentration remaining between 4.1 to 4.9 log CFU/cm^2^. However, the populations of these five isolates did increase from 1.1 to 3.1 log CFU/cm^2^ at 24 h and from 4.1 to 4.8 log CFU/cm^2^ after 72 h at 13 °C.

### 3.4. SEM Observation

**Figure 3:**
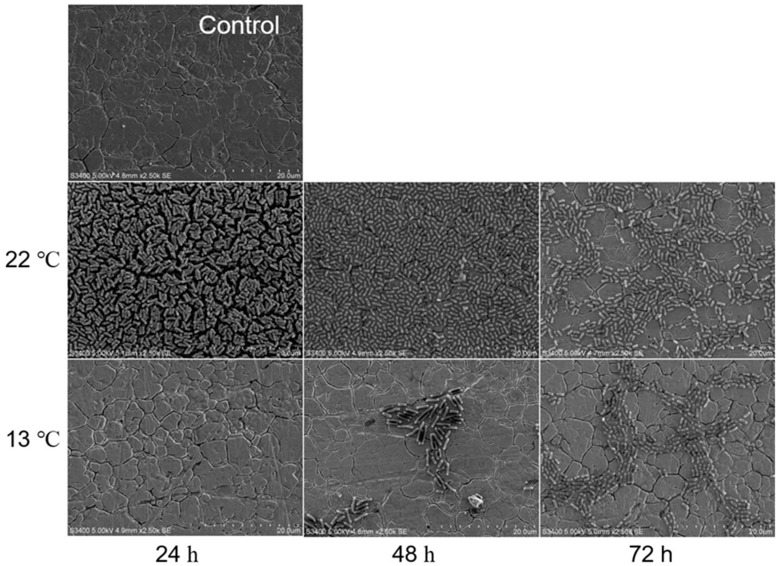
Representative SEM images of O113 biofilm grown in M9 medium at 22 °C and 13 °C for 24, 48, and 72 h on stainless steel coupons. Bar = 20 µm.

**Figure 4:**
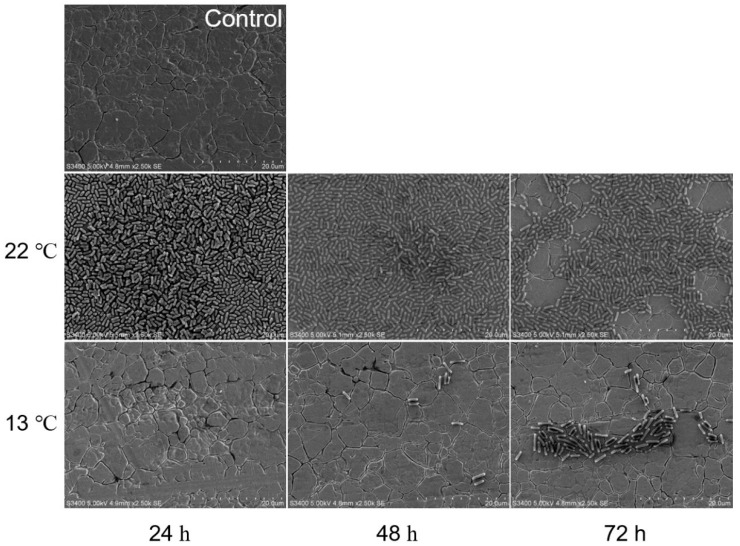
Representative SEM images of O145 biofilm grown in M9 medium at 22 °C and 13 °C for 24, 48, and 72 h on stainless steel coupons. Bar = 20 µm.

**Figure 5:**
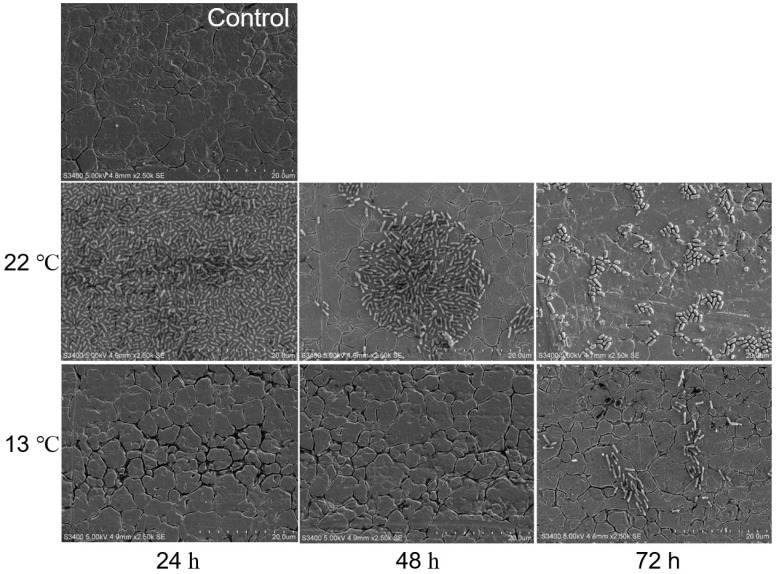
Representative SEM images of O91 biofilm grown in M9 medium at 22 °C and 13 °C for 24, 48, and 72 h on stainless steel coupons. Bar = 20 µm.

Overall, biofilms formed by O113 (Figure 3), O145 (Figure 4), and O91 (Figure 5) at 13 °C were dramatically different from those formed at 22 °C. At 22 °C, the biofilms of strains O113, O145, and O91 consisted of multiple layers of bacterial cells and completely covered the surface of the stainless steel coupon. However, the biofilms decreased with increasing incubation time (Figure 3, Figure 4, and Figure 5). At 72 h, although there were still some cells attached to the surface, they remained in monolayers and were sparsely distributed on the surface. In contrast, no cell aggregates of O113, O145, and O91 were observed on the stainless steel coupon at 13 °C after 24 h, with only sporadic cell aggregates observed on the surface of stainless steel after 72 h.

## 4. Discussion

Biofilms are recognized as one of the major strategies that bacteria utilize to support survival under adverse environmental conditions [25]. Bacteria can form biofilms on a wide variety of surfaces and at air-liquid or liquid-solid interfaces [26,27]. Stainless steel surfaces are widely used in food processing plants [28,29]. However, previous studies have shown that the adhesion of Salmonella to stainless steel was significantly higher than to other materials such as rubber and polyurethane surfaces in processing plants [30]. Stainless steel appears smooth to the unaided eye, but it is actually irregular and can harbor bacterial cells when viewed under a microscope [29].

In this study, the capacity of the 10 STEC isolates varied in their ability to form biofilms on stainless steel (Figure 1A). Similar strain-dependent results were obtained from a study that compared the biofilm-forming ability on polystyrene of 18 O157:H7 strains and 33 non-O157 strains belonging to serogroups O26, O111, O103, and O145 [31]. Another feature of biofilm formation in the current study was that the isolates capable of forming biofilms formed a dense pellicle at the air-liquid interface on stainless steel (white arrows in Figure 1A). It is known that STEC biofilm formation is influenced by a variety of factors, including the characteristics of the strains, nutrient availability, temperature, and other environmental conditions [9]. Under our experimental conditions, the combination of oxygen and moisture available at the air-liquid interfaces may have played an important role in biofilm formation, which is an observation supported by others [20].

Of the 10 isolates that were previously identified as capable of forming strong or moderate biofilms on polystyrene at 22 °C [21], five strains (O113, O145, O91, O157, and O121) were able to form strong or moderate biofilms on stainless steel at this temperature. This indicates that these bacterial isolates can survive on various food-contact surfaces, which increases the likelihood that they could contaminate food. However, the lack of ability for the other five strains (O45, O111, O26, O103, and O128) to form strong biofilms on stainless steel may be due to a number of factors. First, the properties of attachment surface may affect the binding strength of bacteria to the substrate. Second, the differences in the nutrient composition of media might be a contributing factor considering the fact that our previous study was conducted using LB broth [21,32], whereas M9 medium was used to grow the bacteria that formed biofilms in the current study.

At 22 °C, the attached biomass of strains O113, O145, O91, O157, and O121 decreased with increasing incubation time. This reduction in biofilm cells was further confirmed by representative SEM images of O113, O145, and O91 (Figure 3, Figure 4, and Figure 5). Based on our previous studies [21], the biofilms of strains O113, O145, O121, O45, and O103 increased on polystyrene with incubation time, so it was expected that the density of biofilm cells of these strains would also increase on stainless steel over time. Previous studies demonstrated a linear increase in *E. coli* biofilm formation on stainless steel at 23 °C [33] and 15 °C [6] up to 24 h. However, some studies observed a reduction in cell density in biofilms after 48 h [33]. These findings suggest that the biofilm medium plays a major role in the biofilm phenotype. Under static conditions, a lack of nutrients and metabolic waste accumulation in M9 during incubation may contribute to a decrease in biofilm surface populations [5]. Moreover, daughter cells of attached bacteria are often released from the surface upon completion of cell division [34]. These released cells may remain in a planktonic state, which leads to a decrease in STEC biofilm formation. Otherwise, the release of cells from STEC biofilm on stainless steel might have occurred at higher cell densities than during biofilm growth on polystyrene since biofilm cell numbers of STEC continued to increase at 22 °C [21]. Biofilms formed on stainless steel dislodge at a faster rate than those on highly hydrophobic acrylic surfaces [35]. Compared to polystyrene, stainless steel surfaces are hydrophilic and negatively charged at a neutral pH. Since bacteria are also typically negatively charged, this surface may be less conducive to microbial colonization. In weakly charged liquids, the repulsive electrostatic forces are significant [32,36].

Low temperatures (5–15°C) are generally maintained in meat processing environments [37]. Therefore, the potential for STEC strains to develop biofilms on stainless steel at 13 °C was examined. Compared to 22 °C, the biofilm formation of strains O113, O145, O91, O157, and O121 was dramatically inhibited at 13 °C. As previously reported, temperature influences the biofilm-forming capacity of isolates [9]. Weak biofilm formation at lower temperatures could be due to the presence of fewer planktonic cells at 13 °C than at 22 °C (Figure S1). Another explanation could be that biofilm cells grew slower at lower temperatures, which results in weaker biofilms at 13 °C than at 22 °C. These findings are in agreement with Dewanti and Wong [38], who observed stronger biofilm formation by STEC O157:H7 on stainless steel at 22 °C than at 10 °C. Furthermore, the surface properties of STEC at 13 °C may be different from that grown at 22 °C. It has been shown that the cell surface hydrophobicity and fimbriae production of *E. coli* and *Salmonella* are temperature-dependent [39,40,41]. Adrian et al. reported that higher temperature increased the cell surface hydrophobicity level in STEC isolates [40], which is positively correlated with biofilm formation [42,43]. Furthermore, Walker et al. demonstrated that fimbriae in Salmonella were not produced at temperatures below 20 °C [41], which reduced bacterial attachment to commonly used food processing surfaces [44,45]. We also found that, over 72 h of incubation, only strain O113 formed a moderate biofilm at 13 °C. This biofilm-forming capacity of O113 may contribute to its persistence in the processing environment and influence its high relative incidence [46].

## 5. Conclusions

The findings in this study indicated that STEC isolates form biofilms in a strain-dependent manner and the process was affected by various environmental factors such as temperature, atmosphere, and incubation time. Of the ten isolates previously shown to readily form biofilms on polystyrene, only strains O113, O145, O91, O157, and O121 formed moderate to strong biofilms on stainless steel at 22 °C. At 13 °C, biofilm formation by strains O113, O145, O91, O157, and O121 decreased, which indicates that low temperature environments will reduce STEC biofilm formation on food contact surfaces. Further studies are underway to assess the ability of these strains to form biofilms in meat processing environments and to identify methods to remove and prevent biofilm development as a means of reducing the risk of food contamination.

## Supplementary Materials

The following are available online at https://www.mdpi.com/2076-2607/7/4/95/s1.
