# Supplementary material for: Biofilm Formation by Shiga Toxin-Producing Escherichia coli on Stainless Steel Coupons as Affected by Temperature and Incubation Time
Source: Microorganisms. 2019 Mar 31;7(4):95. doi: 10.3390/microorganisms7040095 (PMC6518284; doi:10.3390/microorganisms7040095)
Supplement: Supplementary file 1 [file microorganisms-07-00095-s001.pdf]

microorganisms

Supplementary

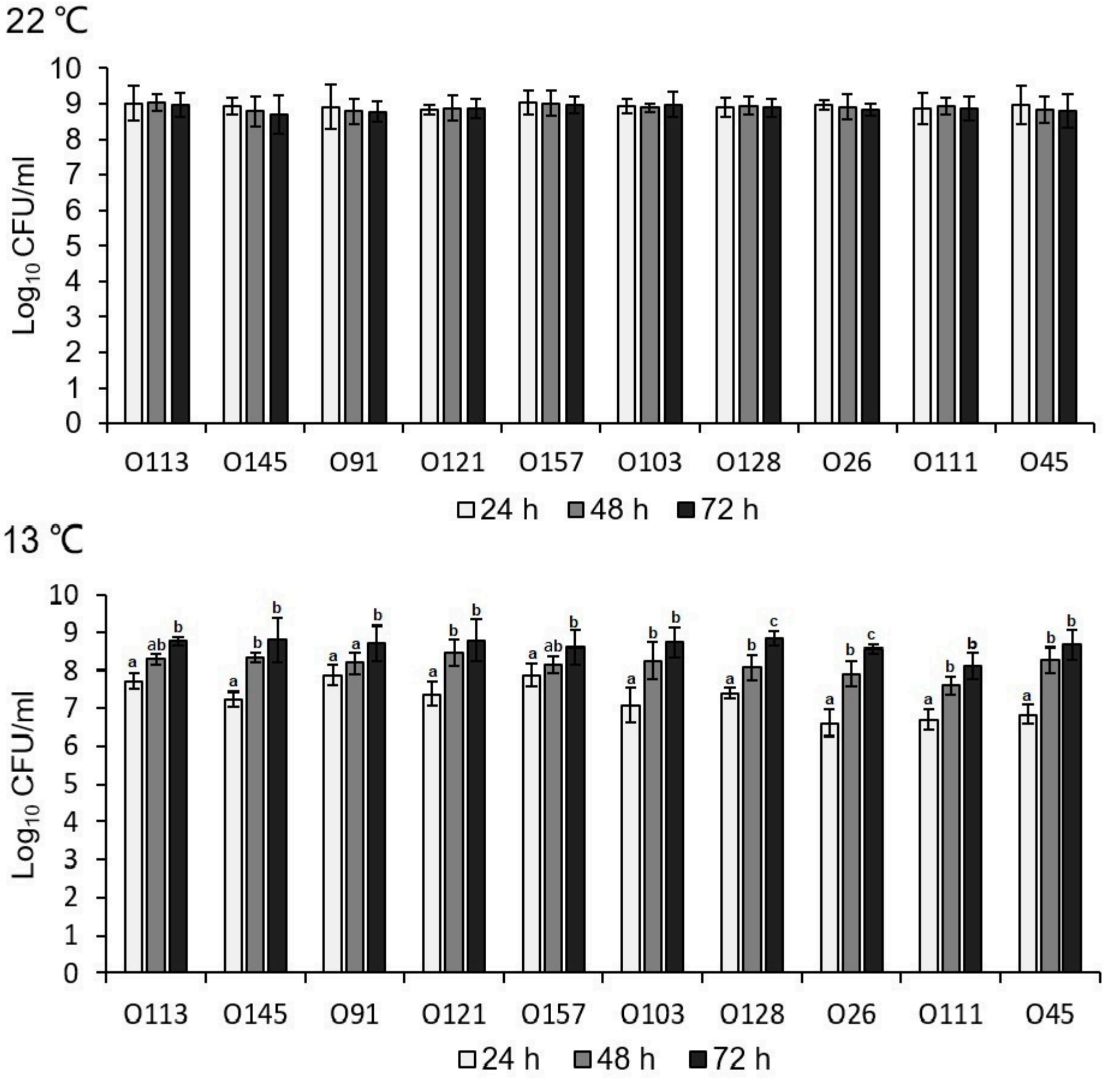

Figure 1. Growth of planktonic cells of the 10 STEC isolates after incubation at 22 °C (A) or 13 °C (B) for 24, 48 and 72 h. Means within a temperature and a strain with different superscripts differ ($P < 0.05$).
